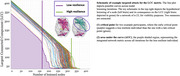# Resting‐State Network Metrics of Brain Resilience to Targeted Attack

**DOI:** 10.1002/alz70856_102684

**Published:** 2025-12-25

**Authors:** Georgette Argiris, Yaakov Stern, Christian G Habeck, Eider M Arenaza‐Urquijo, Gabriele Cattaneo, Javier Solana‐Sánchez, Alvaro Pascual‐Leone, María Cabello‐Toscano, David Bartrez‐Faz

**Affiliations:** ^1^ Columbia University Irving Medical Center, New York, NY, USA; ^2^ Taub Institute for Research in Alzheimer's Disease and the Aging Brain, Columbia University, New York, NY, USA; ^3^ Cognitive Neuroscience Division, Columbia University, New York, NY, USA; ^4^ Taub Institute for Research on Alzheimer's Disease and the Aging Brain, Columbia University, New York City, NY, USA; ^5^ ISGlobal ‐ Barcelona Institute for Global Health, Barcelona, Catalunya/Barcelona, Spain; ^6^ Guttmann Brain Health Institute, Institut Guttmann, Institut Universitari de Neurorehabilitació Adscrit a la UAB., Badalona, Barcelona, Spain; ^7^ Institut Guttmann, Institut Universitari de Neurorehabilitaci. adscrit a la Universitat Aut.noma de Barcelona, Barcelona, Spain, Barcelona, Barcelona, Spain; ^8^ Hinda and Arthur Marcus Institute for Aging Research, and Deanna and Sidney Wolk Center for Memory Health, Hebrew Senior Life, Boston, MA, USA; ^9^ Department of Medicine, Faculty of Medicine and Health Sciences and Institute of Neurosciences, University of Barcelona, Barcelona, Barcelona, Spain; ^10^ Department of Medicine, Faculty of Medicine and Health Sciences and Institute of Neurosciences, University of Barcelona, Barcelona, Spain, Barcelona, Barcelona, Spain

## Abstract

**Background:**

Recent advancements in brain network analyses enable more precise measurements of network integrity, including resilience to perturbations affecting functionality. Identifying neural markers of cognitive reserve (CR) is crucial for understanding individual differences in age‐related cognitive changes. In a previous study of cognitive healthy older adults, we identified a measure of network resilience that moderated the effect of brain integrity on longitudinal cognitive performance (Argiris et al., 2024). In the current study, we applied this approach to a different cohort and tested additional network metrics as potential mechanisms of CR.

**Method:**

Five hundred sixty‐nine cognitively healthy participants from the Barcelona Brain Health Initiative (BBHI) cohort (ages 42–67) underwent resting‐state fMRI to measure functional connectivity and neuropsychological testing at baseline and ∼2.5 years later. Using the Schaefer et al. (2018) 400‐parcellation atlas plus 19 subcortical regions, undirected weighted adjacency matrices (419 regions total) were generated. Resilience metrics were extracted at follow‐up, reflecting recent brain status for CR quantification.

A targeted attack approach sequentially removed nodes based on strength, extracting metrics at each iteration: (1) largest connected component (LCC), (2) characteristic path length, and (3) clustering coefficient. The (a) critical point of drop and (b) area under the curve (AUC) were evaluated. Resilient individuals were expected to sustain larger LCCs over more iterations, with higher clustering coefficients and lower characteristic path lengths hypothesized as mechanisms of CR. Network metrics were tested for their ability to predict cognitive performance changes or moderate cortical thickness (CT) effects, adjusting for demographic factors, baseline variables, and unlesioned matrix metrics.

**Result:**

The critical point of characteristic path length significantly predicted cognitive decline on the WAIS and TMT, with higher resilience associated with slower decline. A higher AUC of clustering coefficient predicted better WAIS outcomes. No effects were found for LCC or moderation of CT by brain resilience metrics.

**Conclusion:**

Our findings support targeted attack metrics as measures of CR, where higher resilience in network metrics predicted cognitive outcomes beyond structural brain changes. Future research should explore network metrics as protective factors against age‐related changes, aligning with the Resilience and Reserve consortium framework (Stern et al., 2023).